# Cavernous Sinus Thrombosis due to Chronic Bacterial Sinusitis

**DOI:** 10.7759/cureus.4712

**Published:** 2019-05-21

**Authors:** Summia Matin Afridi, Myint Noe, Ahmad Raja, Akriti G Jain

**Affiliations:** 1 Internal Medicine, Florida Hospital, Orlando, USA; 2 Internal Medicine, Presence Saint Francis Hospital, Evanston, USA

**Keywords:** cavernous sinus thrombosis, bacterial sinusitis, diabetes mellitus complications

## Abstract

The cavernous sinus is the most frequent dural sinus to become infected and thrombosed. Septic cavernous sinus thrombosis has become rare since the advent of antibiotics. We herein present a case of septic cavernous sinus thrombosis caused by chronic bacterial sinusitis.

## Introduction

The cavernous sinus is the most frequent dural sinus to become infected and thrombosed [1]. It is most centrally located of the dural sinuses, positioned just lateral to the base of the sella turcica and to the sphenoid air sinuses. It contains multiple trabeculae which can trap bacteria, thus making the risk of infection higher as compared with other sinuses. There are three common sites of infection which can spread to cavernous sinus, one of which is from paranasal sinuses that includes ethmoid, maxillary and sphenoid sinuses. The infection can spread to the cavernous sinus through the emissary veins or break through the lateral sinus wall [1, 2]. Septic cavernous sinus thrombosis has become rare since the advent of antibiotics. We herein present a case of septic cavernous sinus thrombosis caused by chronic bacterial sinusitis.

## Case presentation

**Figure 1:**
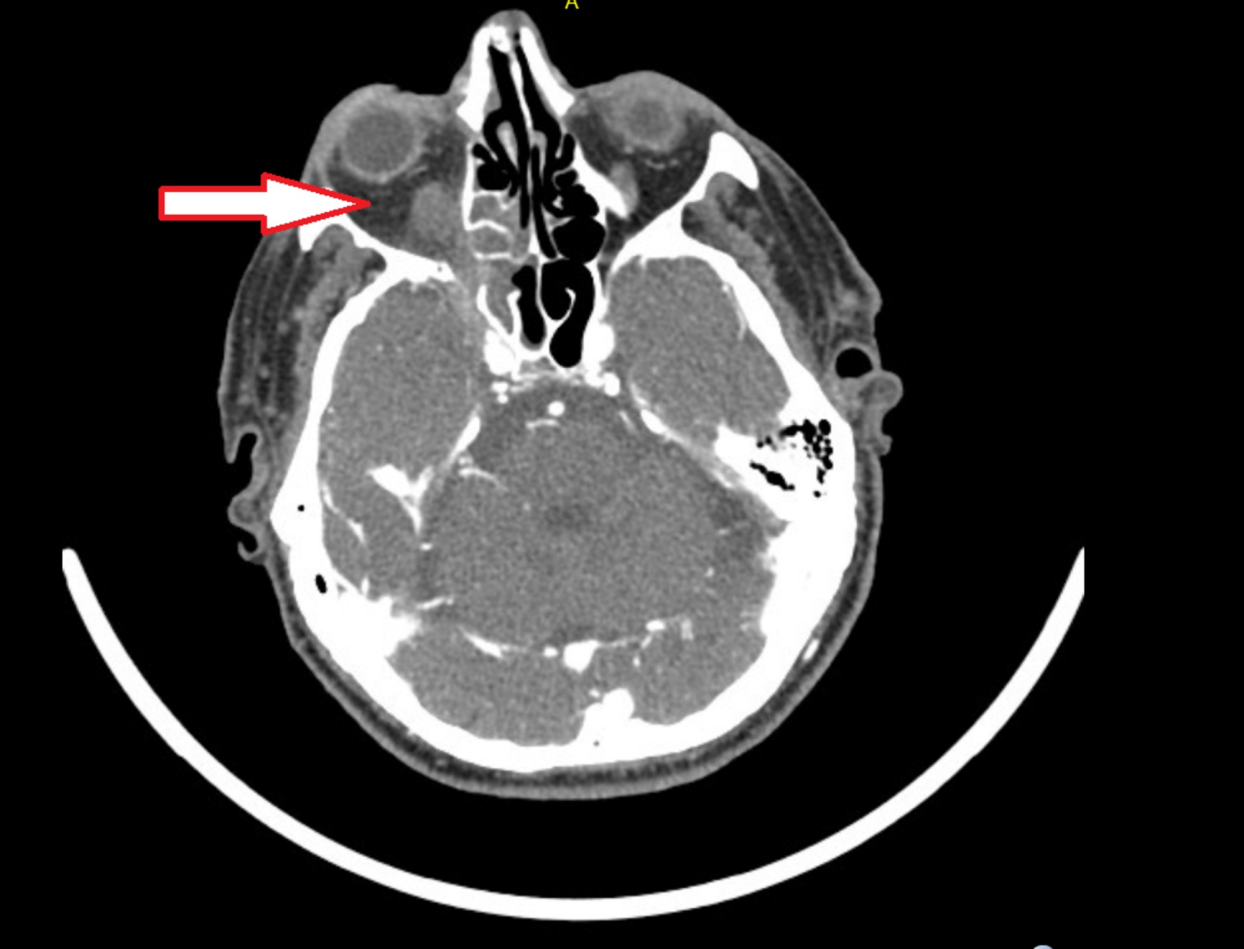
CT head concerning for invasive fungal sinusitis with abnormal soft tissue infiltrating the orbital apex (arrow).

**Figure 2:**
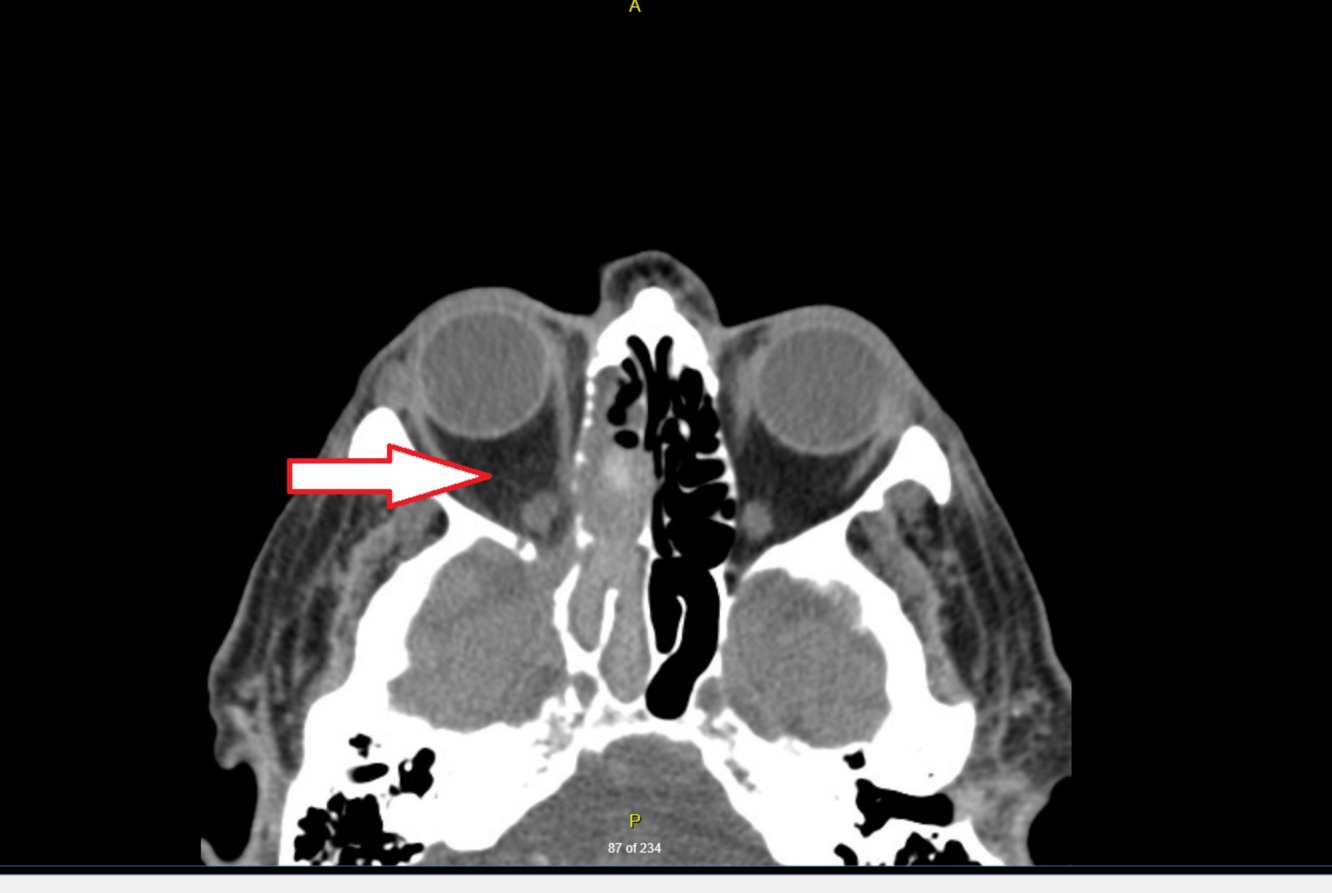
CTA head consistent with invasive fungal sinusitis involving the right paranasal sinuses (arrow). CTA: Computed tomography angiography

A 77-year-old diabetic patient, with end-stage renal disease, presented to emergency department with three-month history of gradually worsening right-sided headache and visual loss. The patient had an extensive workup outside the hospital including temporal artery biopsy, CT angiogram of the head and neck which were all reportedly negative. On initial examination, the patient had right-sided ptosis, ophthalmoplegia and a non-reactive pupil. The differential diagnosis included migraine, glaucoma, cavernous sinus thrombosis due to visual loss, stroke, and invasive fungal sinusitis due to history of underlying diabetes mellitus. Meningitis and encephalitis were not high in the differential due to lack of fever, confusion and signs of meningismus on physical examination. On repeat CT head (Figure 1), CT angiogram and venogram of the brain (Figure 2) showed findings consistent with invasive fungal sinusitis involving the right paranasal sinuses with large areas of bony dehiscence involving the right sphenoid sinus, ethmoid air cells, and right maxillary sinus.

There was an abnormal soft tissue infiltrating the orbital apex encasing the optic nerve sheath complex along with dehiscence of the optic canal. The images also showed partial thrombosis of the right anterior cavernous sinus. Therefore, the patient was immediately started on liposomal amphotericin B as well as broad-spectrum antibiotic coverage with vancomycin and cefepime. The patient then underwent debridement and resection of posterior ethmoid, orbital apex lesion, decompression of the medial orbital wall, optic canal and optic nerve with expanded endonasal approach. During the procedure, purulent material was found suggestive of bacterial infection and there was no evidence of dark necrotic tissue or dead mucosa. There was apparent erosion and absence of the bony medial orbital wall posteriorly toward the orbital apex. Samples were taken and sent for microbiology and histopathology. Pseudomonas aeruginosa, Staphylococcus aureus and Staphylococcus epidermidis were isolated from the surgical culture. Both cultures and pathology were not supportive of fungal infection and hence, amphotericin B was stopped. The patient was also anticoagulated with heparin which was then switched to apixaban. During the hospital stay, headache resolved with no change in his visual acuity. He was later discharged to inpatient rehabilitation facility and completed four weeks of intravenous antibiotics.

## Discussion

The mainstay of therapy for septic cavernous sinus thrombosis is antibiotics. Before the use of antimicrobial agents, mortality was near 100%, but it markedly decreased to approximately 20% to 30% during the antibiotic era [3, 4]. High dose intravenous antibiotics should be started emergently directed against the most probable organism. The initial antibiotics should cover methicillin-resistant Staphylococcus aureus (MRSA) with vancomycin which can be later switched to nafcillin or oxacillin if cultures reveal methicillin sensitive staphylococcus. This should be combined with a third or fourth generation cephalosporin, either ceftriaxone (2 g IV every 12 hours) or cefepime (2 g IV every 8 to 12 hours); the latter agent is preferred if patient is a risk for pseudomonas. A minimum of three weeks of treatment with antibiotics is usually required.

Surgery is appropriate in selected cases as debridement of the sinuses shows rapid improvement [5]. The use of catheterization and thrombectomy is an option for patients with rapidly worsening neurological symptom despite adequate heparin therapy.

Although it is still controversial with limited data to support, there were evidences suggesting reduced morbidity with the early use of anticoagulation in combination with the antibiotics. Constant infusion heparin anticoagulation should be used, adjusting the dose to maintain a partial thromboplastin time ratio of 1.5 to 2.5 [6]. In one case series, patients with cavernous sinus thrombosis who received anticoagulation did not have increased incidence of hemorrhagic complications especially in patients without underlying central nervous system infection. It indicates that it can be considered safe to add anticoagulation to antibiotics and surgery in the management of patients with cavernous sinus thrombosis [7].

There is a lack of evidence to support the role of glucocorticoids in the management of septic cavernous sinus thrombosis. And an important side effect of glucocorticoids is immunosuppression which can be detrimental in setting an active infection.

There have been several cases of cavernous sinus thrombosis reported due to underlying bacterial sinusitis. In one literature review, 88 such case reports were identified over a period of 25 years (between January 1980 and July 2015) with the most common organism being found was MRSA. Fungal infections were also seen but they were more common in immunosuppressed patients, for example, patients with underlying diabetes mellitus, immunosuppressive medication use and hematological malignancies [8]. In another retrospective study of seven patients with cavernous sinus thrombosis, sphenoid sinus was found to be involved in all these patients. In addition, headache (100%) and cranial nerve involvement (86%) were the two most common presenting symptoms along with fever (71%) and orbital symptoms (71%) [9]. In addition to cavernous sinus thrombosis, other serious complications of bacterial sinusitis like cavernous sinus thrombophlebitis have also been reported [10].

In our case, even though the patient was immunosuppressed with underlying diabetes mellitus and end-stage renal disease with increased risk of fungal infections, he was found to have underlying bacterial sinusitis which lead to the complication of cavernous sinus thrombosis. In addition, the presentation of cavernous sinus thrombosis can be similar to the presentation of other causes of headache. For example, in our case the patient was initially suspected to have giant cell arteritis and underwent treatment and extensive workup for that which could have contributed to the delay in diagnosis and start of appropriate treatment with antimicrobial agents. Therefore, a high clinical suspicion, thorough history, detailed physical examination and appropriate diagnostic tests are usually required for the diagnosis of this condition.

## Conclusions

In conclusion, the overall mortality rate associated with septic cavernous sinus thrombosis is 30%. The infection can spread to meninges and pituitary gland leading to meningitis and pituitary insufficiency respectively despite appropriate management. About 30% of patients can develop complications like persistent oculomotor weakness, blindness and hemiparesis. Timely diagnosis and prompt management is crucial for good outcome. Clinicians should recognize this fatal complication and initiate appropriate treatment of sinusitis before the infection spreads.
